# The role of gut microbiota in paediatric short bowel syndrome

**DOI:** 10.3389/fcimb.2026.1970119

**Published:** 2026-10-07

**Authors:** Cheng Zeng, Lidan Liang, Tingfang Lv

**Affiliations:** 1 Department of Paediatrics, Luzhou People’s Hospital, Luzhou, China; 2 Department of Paediatrics, Luxian People’s Hospital, Luzhou, China; 3 Department of Paediatrics, Children’s Medical Center, The Affiliated Hospital of Southwest Medical University, Luzhou, China; 4 Sichuan Clinical Research Center for Birth Defects, Luzhou, China

**Keywords:** dysbiosis, intestinal failure, microbiome, pediatric, short bowel syndrome

## Abstract

Short bowel syndrome (SBS) is one of the leading causes of intestinal failure in children. Its development is influenced not only by the length and function of the residual intestine but is also closely related to the homeostasis of the gut microbiota. In recent years, the role of the gut microbiota in intestinal adaptation, metabolic regulation, and the development of complications in pediatric SBS has gradually garnered attention. This review summarizes the characteristics of the gut microbiota, mechanisms underlying dysbiosis, associations with complications, and microbiota-targeted therapeutic strategies in pediatric SBS. Existing studies indicate that children with SBS exhibit reduced microbiota diversity, compositional abnormalities, and metabolic dysfunction. Gut microbiota dysbiosis may be associated with intestinal barrier disruption, immune dysregulation, alterations in residual intestinal structure, and parenteral nutrition, and may contribute to the development and progression of complications such as small intestinal bacterial overgrowth (SIBO), D-lactic acidosis (D-LA), and liver injury. Strategies such as probiotics, nutritional modulation, and fecal microbiota transplantation (FMT) have shown potential clinical value, although their efficacy and safety require further validation. Future studies integrating multi-omics approaches and long-term longitudinal investigations are needed to elucidate the mechanisms underlying interactions between the microbiota and host, thereby providing a theoretical basis for microbiota-targeted interventions and improving long-term outcomes in children with SBS.

## 1 Introduction

SBS results from the loss of effective intestinal length and absorptive function due to extensive resection of the small intestine or intestinal developmental abnormalities, and is one of the leading causes of intestinal failure in children (O'Keefe et al., 2006). Pediatric SBS most commonly develops secondary to conditions such as necrotizing enterocolitis, intestinal atresia, and intestinal volvulus (Channabasappa et al., 2020). Affected children may present with diarrhea, electrolyte disturbances, and impaired nutrient absorption, with severe cases requiring long-term dependence on parenteral nutrition (PN) (Neelis et al., 2019). Although nutritional support and surgical treatment have improved survival rates in these children, complications such as PN-associated liver disease, catheter-related infections, SIBO, and growth retardation continue to have a long-term impact on prognosis (Cole et al., 2010).

Intestinal adaptation is a key process by which children with SBS gradually reduce and ultimately overcome their dependence on PN (Premkumar et al., 2022). Following intestinal resection, the remaining intestine undergoes structural remodeling, including villus elongation, crypt deepening, and expansion of absorptive surface area, thereby enhancing nutrient absorption capacity (Bering and DiBaise, 2023). Recent studies, including pediatric SBS cohorts and complementary experimental studies, suggest that intestinal adaptation involves not only host tissue remodeling but also interactions with the gut microbiota, immune responses, and microbial metabolites (De Meyere et al., 2026). The gut microbiota participates in nutrient metabolism, short-chain fatty acid (SCFA) production, maintenance of the mucosal barrier, and immune regulation. The interaction between the host and gut microbiota may represent an important regulatory mechanism influencing intestinal adaptation in SBS (Adak and Khan, 2019).

Compared with healthy children of the same age, children with SBS typically exhibit reduced microbial diversity, delayed microbiota maturation, an increased abundance of potentially pathogenic bacteria represented by Proteobacteria, and a decreased abundance of beneficial bacteria with metabolic functions (Davidovics et al., 2016; Carter et al., 2023; Vaou et al., 2026). In children with SBS, Gut microbiota dysbiosis may exacerbate impaired nutrient absorption by increasing intestinal permeability, disrupting the mucosal barrier, and inducing inflammatory responses. It may also further interfere with intestinal adaptation through alterations in microbial metabolites (Till et al., 2015; Zeichner et al., 2019). Furthermore, microbiota dysbiosis is closely associated with SIBO, bacteremia, D-LA and intestinal failure-associated liver disease (IFALD) (Wang et al., 2022).

In recent years, advances in multi-omics technologies have facilitated research on the gut microbiota in children with SBS, suggesting that the microbiota may contribute to intestinal adaptation and complication development. However, previous reviews have generally addressed SBS or intestinal failure from a broader perspective, with limited emphasis on pediatric-specific evidence. This article provides a narrative review of the gut microbiota in children with SBS, integrating microbiota alterations with residual intestinal anatomy, nutritional exposure, and major SBS-related complications. It also critically evaluates microbiota-related interventions and highlights clinically relevant priorities for future research, providing a more focused and evidence-based perspective on pediatric SBS.

## 2 Literature search strategy

This narrative review searched PubMed, Embase, and Web of Science databases up to July 2026. The search terms mainly included “short bowel syndrome,” “intestinal failure,” “pediatric,” “children,” “infants,” “gut microbiota,” “gut microbiome,” “dysbiosis,” “intestinal adaptation,” “small intestinal bacterial overgrowth,” “short-chain fatty acids,” “bile acids,” “intestinal failure-associated liver disease,” and “probiotics,” with modifications according to the specific search strategies of each database. Original clinical studies, observational studies, intervention studies, and relevant animal experiments addressing SBS, intestinal adaptation, gut microbiota, microbial metabolites, or microbiota-related complications were considered. Pediatric and adolescent studies were prioritized. Adult SBS studies, studies of intestinal failure, and preclinical studies were considered when pediatric evidence was limited or when they provided relevant mechanistic information. Reviews and key publications were used to provide background and contextual information. Studies unrelated to SBS or intestinal failure, studies without relevant microbiota-related outcomes, and publications lacking sufficient information for interpretation were excluded.

## 3 Gut microbiota characteristics in children with SBS

### 3.1 Alterations in gut microbial composition and diversity

In children with SBS, studies of fecal and intestinal microbiota have reported substantial dysbiosis, including reduced microbial richness and diversity, depletion of some beneficial or SCFA-producing taxa, and expansion of potentially pathogenic microorganisms (Norsa et al., 2019). Compared with age-matched healthy children, children with SBS show increased relative abundances of *Enterobacteriaceae*, *Escherichia*, and *Klebsiella*, along with decreased abundances of *Bacteroidetes*, *Bifidobacterium*, and several beneficial SCFA-producing bacteria (Huang et al., 2017). Studies have demonstrated that reduced beneficial bacteria, increased pro-inflammatory *Enterobacteriaceae*, and impaired microbial functions are associated with gut dysbiosis in children with SBS, and the severity of microbiota alterations correlates with growth impairment (Piper et al., 2017). Furthermore, children with shorter residual small bowel lengths exhibit more pronounced reductions in microbial diversity, enrichment of *Proteobacteria*, and prolonged dependence on PN, suggesting that intestinal anatomical factors may influence intestinal microbial composition and maturation (Engelstad et al., 2018). The gut microbiota of children with SBS may also exhibit delayed maturation, although this observation is difficult to distinguish from the effects of prematurity, intestinal resection, and prolonged hospitalization (Ihekweazu and Versalovic, 2018).

Gut microbiota characteristics are also closely associated with intestinal adaptation in children with SBS. Longitudinal studies have shown that children with higher baseline microbial diversity and lower abundance of *Enterobacteriaceae* are more likely to achieve PN independence, whereas persistent enrichment of *Proteobacteria* is associated with impaired intestinal adaptation (Engstrand Lilja et al., 2015). Microbiota dysbiosis is also closely linked to SBS-related complications. Children with IFALD or central line-associated bloodstream infection (CLABSI) exhibit reduced microbial diversity, accompanied by expansion of *Enterobacteriaceae* and decreased acetate levels (Wang et al., 2017). Notably, the gut microbiota of children with SBS demonstrates dynamic changes over time. With increased enteral nutrition, reduced PN dependence, and gradual recovery of intestinal function, certain microbial characteristics may shift toward a healthier profile (Cleminson et al., 2025). Therefore, gut microbiota alterations in pediatric SBS represent a dynamic process rather than a fixed abnormality, and are closely intertwined with disease severity, intestinal adaptation, and therapeutic progression.

### 3.2 Functional and metabolic alterations of the gut microbiota

SBS not only alters gut microbial composition but also induces functional remodeling and metabolic disturbances, including disruptions in pathways related to SCFA production, bile acid transformation, and amino acid metabolism (Josyabhatla and Imseis, 2021). Among these alterations, a marked reduction in key anaerobic SCFA-producing bacteria and decreased levels of SCFAs, such as butyrate and propionate, have been consistently observed (Wolfschluckner et al., 2023). Moreover, the abundance of SCFA-producing bacteria is inversely associated with PN requirements in children with intestinal failure, suggesting that microbial metabolic functions may contribute to intestinal adaptation and the regulation of nutritional dependence (Cerdó et al., 2022). Meanwhile, abnormalities in bile acid and amino acid metabolism associated with SBS may further disrupt intestinal–hepatic metabolic homeostasis. Animal studies have shown that SBS-associated microbiota dysbiosis primarily involves alterations in *Firmicutes* and *Bacteroidetes*, accompanied by disturbances in amino acid and branched-chain amino acid metabolism, suggesting that microbiota remodeling may contribute to SBS-related liver injury through microbiota–host metabolic interactions (Pereira-Fantini et al., 2017). In addition, SBS children with growth impairment exhibit more severe functional abnormalities of the gut microbiota, including reduced abundance of beneficial SCFA-producing anaerobes, expansion of pro-inflammatory *Enterobacteriaceae*, and alterations in metabolic pathways related to amino acid metabolism and the tricarboxylic acid cycle (Piper et al., 2017).

Functional and metabolic disturbances of the gut microbiota may also may contribute to SBS-related complications through intestinal barrier disruption and immune activation. Reduced SCFA levels can impair epithelial energy supply and maintenance of tight junction integrity, while microbial dysbiosis is associated with the accumulation of harmful metabolites such as endotoxins, increase bacterial translocation, and induce the release of inflammatory cytokines, including IL-6 and TNF-α (Hajam et al., 2021). These alterations are associated with intestinal barrier dysfunction and systemic inflammation, and may affect hepatic metabolism and inflammatory responses through the gut–liver axis, thereby potentially increasing the risk of complications such as PN-associated infections (Zhang et al., 2021). Therefore, gut microbiota alterations in SBS represent more than simple compositional changes, involving systemic disturbances in microbial metabolic functions, microbial-derived metabolites, and host immune-metabolic networks, which may collectively influence disease progression.

In summary, gut microbiota alterations in children with SBS involve not only changes in intestinal microbial composition and diversity but also functional and metabolic dysregulation. These abnormalities may contribute to growth impairment and PN-associated complications by affecting intestinal barrier integrity and host immune-metabolic responses. Because most studies rely on fecal samples, their findings may not fully represent the microbiota of the small intestine, particularly in SBS with altered intestinal anatomy, rapid transit, or SIBO. The major characteristics of the gut microbiota and related findings in children with SBS are summarized in **Table 1**.

**Table 1:** Clinical and microbiome characteristics of studies investigating gut microbiota in pediatric SBS and intestinal failure.

Study design	Subjects and sample size	Age range	Sampling site/specimen	Methods	Findings	Major limitations	Ref.
Observational study	Children with SBS (n=9) and healthy controls (n=8)	4 months-8 years	Feces	16S rRNA sequencing	SBS children showed distinct microbiota composition, with increased Proteobacteria and *Lactobacillus* and reduced *Ruminococcus* abundance	Small sample size; limited generalizability	(Davidovics et al., 2016)
Observational study	Children with SBS (n=12) and healthy controls (n=5)	<16 years	Feces	16S rRNA sequencing; fecal SCFA analysis	Reduced SCFA-producing anaerobes, decreased butyrate levels, and increased relative abundance of *Escherichia coli* were observed in SBS	Small sample size; observational design	(Dowhaniuk et al., 2020)
Observational study	Infants with SBS (n=18) and healthy controls (n=7)	2–9 months	Feces	16S sequencing; SCFAs, bile acids and sIgA	Infants with IFALD and CLABSI exhibited reduced microbial diversity, *Enterobacteriaceae* enrichment, and decreased acetate levels	Small infant cohort; potential effects of prematurity, nutrition and antibiotics	(Wang et al., 2017)
Observational study	Infants with SBS (n=7) and healthy controls (n=11)	2–13 months	Ostomy effluent and feces	16S rRNA sequencing	SBS infants showed increased *Proteobacteria* and *Enterococcus*, reduced SCFA-producing bacteria, and impaired microbial function	Small sample; sampling-site differences may affect microbiota profiles	(Zhang et al., 2020)
Observational study	Infants with SBS (n=15) and healthy controls (n=3)	<2 years	Small intestine and feces	16S rRNA sequencing; qPCR	SBS infants exhibited marked alterations in microbial diversity and composition, with distinct profiles between small intestine and feces	Very small control group; cross-sectional design	(Piper et al., 2020b)
Observational study	Children with intestinal failure (n=44, 70% with SBS)	Median 58 months	Multiple intestinal sites/specimens	Culture and metagenomic sequencing	Children with SIBO showed reduced α-diversity, enrichment of *Enterobacteriaceae* and anaerobes, and associations with liver injury and infections	Mixed intestinal-failure population; not all participants had SBS	(Hilberath et al., 2025)
Observational study	Children with SBS (n=25) and healthy controls (n=4)	2–10 years	Jejunal and feces	16S rRNA sequencing	SBS children showed reduced microbial diversity, with more severe dysbiosis associated with PN dependence, shorter residual bowel length, and absence of ileocecal valve	Very small control group; cross-sectional design	(Zeichner et al., 2019)

IF, intestinal failure; PN, parenteral nutrition; CLABSI, central line-associated bloodstream infection; IFALD, intestinal failure-associated liver disease; SIBO, small intestinal bacterial overgrowth.

## 4 Potential mechanisms underlying gut microbiota dysbiosis in pediatric SBS

### 4.1 Intestinal barrier dysfunction and immune regulation

Gut microbiota dysbiosis in children with SBS is closely associated with impaired intestinal mucosal barrier function. The intestinal barrier is composed of the mucus layer, antimicrobial peptides, secretory immunoglobulin A, and commensal microbiota, which collectively prevent the translocation of pathogenic microorganisms into the mucosa (Vancamelbeke and Vermeire, 2017). Following intestinal resection, reduced mucosal surface area, accelerated intestinal transit, and insufficient local nutrient stimulation may impair epithelial integrity and barrier function (Courtney et al., 2021). Meanwhile, bacterial overgrowth and alterations in intestinal microbial composition may increase exposure to microbial components such as endotoxins, further disrupting epithelial barrier integrity (DeGonza et al., 2025). In children with SBS, reductions in SCFA-producing bacteria have been associated with altered intestinal barrier function. Experimental studies further suggest that SCFAs can support epithelial energy metabolism and tight-junction integrity, providing a potential mechanistic explanation for this association (Ruiz et al., 2026). Furthermore, in infants with SBS, the absence of the ileocecal valve may weaken the barrier function between the small intestine and colon, potentially facilitating the migration of colonic microbiota into the proximal small intestine and increasing the risk of bacterial overgrowth and mucosal injury (Peters et al., 2022).

Microbiota dysbiosis may further exacerbate intestinal inflammation through immune activation. Experimental and broader microbiome studies suggest that SCFA-producing bacteria can promote regulatory T-cell responses and anti-inflammatory signaling. In children with SBS, however, direct clinical evidence linking depletion of these taxa to impaired Treg-mediated immune regulation remains limited (Barbara et al., 2021). Following microbiota disruption, reduced SCFA production may weaken these immunomodulatory effects and consequently impairs Treg-mediated immune homeostasis (Turato et al., 2016). Experimental studies have shown that abnormal expansion of Gram-negative bacteria and increased exposure to lipopolysaccharide (LPS) may activate pattern recognition receptors, such as Toll-like receptor 4 (TLR4), on intestinal epithelial cells, thereby triggering inflammatory signaling pathways including NF-κB and promoting the release of pro-inflammatory cytokines such as IL-6, TNF-α, and IL-1β (Sanaksenaho et al., 2021). In addition, disruption of the intestinal barrier may facilitates bacterial translocation across the mucosa, resulting in persistent activation of local and systemic immune responses (Barron et al., 2017). These alterations interact with each other to further exacerbate intestinal barrier damage and immune dysregulation, thereby affecting intestinal adaptation and increasing the risk of infections and SBS-associated complications.

### 4.2 Alterations in residual intestinal structure and function

The anatomical characteristics and functional status of the residual intestine are important determinants of gut microbiota composition in children with SBS. Residual bowel length directly influences the effective absorptive surface area and intestinal transit of luminal contents (Goulet et al., 2005). Shorter residual bowel length limits nutrient absorption and alters the intestinal luminal environment, potentially increasing the risk of microbiota dysbiosis. In children with SBS, studies have shown that those with residual small bowel length <35 cm exhibit increased relative abundance of *Proteobacteria*, accompanied by greater PN dependence and poorer growth outcomes (Engelstad et al., 2018). In addition to bowel length, preservation of the ileocecal valve, colonic continuity, and stoma status can also modify the intestinal microenvironment (Saker et al., 2026). Loss of the ileocecal valve may reduce the barrier against retrograde migration of colonic microbiota, whereas alterations in intestinal continuity and the presence of a stoma can further influence microbial colonization patterns. A study investigating SBS patients with different anatomical subtypes demonstrated distinct microbiota profiles among SBS types; SBS type II was characterized by enrichment of *Proteobacteria* and depletion of *Firmicutes* and *Bacteroidetes*, whereas SBS type III showed increased relative abundance of *Lactobacillus* and *Prevotella* (Huang et al., 2017). Furthermore, impaired intestinal motility, anastomotic strictures, and localized bowel dilation may promote luminal content stasis, creating favorable conditions for bacterial colonization and overgrowth (Mangalat et al., 2026). Therefore, the anatomical and functional characteristics of the residual intestine may contribute to the development of characteristic gut microbiota dysbiosis in children with SBS by reshaping the intestinal luminal environment.

### 4.3 Effects of nutritional support and antibiotic exposure on gut microbiota

Nutritional support strategies are important modifiable factors influencing gut microbiota composition in children with SBS. Long-term PN or insufficient enteral nutrition may reduce luminal nutrient stimulation, impairs intestinal mucosal adaptation, affects tight junction integrity, and decreases the availability of fermentable substrates such as dietary fiber, thereby limiting the maintenance of SCFA-producing bacteria and potentially promoting the colonization of opportunistic microorganisms, including *Proteobacteria* (Buchman et al., 1995; Cerdó et al., 2022). Studies have shown that a higher proportion of energy derived from PN is associated with reduced intestinal α-diversity (Talathi et al., 2023). In contrast, early initiation of appropriate enteral nutrition can increase luminal nutrient supply, promote intestinal adaptation, and support microbial homeostasis. Therefore, when clinically feasible, gradual advancement of enteral nutrition and reduction of PN dependence may help restore intestinal microbial balance (Neelis et al., 2022). Long-term PN is also closely associated with complications such as IFALD and may further influence intestinal microbial composition and host metabolic homeostasis through the gut–liver axis (Pierret et al., 2019).

Antibiotic and other medication exposures represent additional important iatrogenic factors associated with gut microbiota dysbiosis in children with SBS (Phyo et al., 2021). Due to the increased risk of SIBO, catheter-related infections, and other infectious complications, children with SBS frequently require repeated or prolonged antibiotic treatment (Talathi et al., 2021). Studies have demonstrated that beneficial bacteria, including *Bifidobacterium* and *Lactobacillus*, are markedly depleted or nearly absent in the intestinal microbiota of antibiotic-exposed children with SBS, whereas resistant organisms such as *Enterococcus* and *Klebsiella* are significantly enriched (Thänert et al., 2021). Broad-spectrum antibiotics can non-selectively eliminate commensal microorganisms, reduce microbial diversity, and consequently increase the risk of colonization by resistant bacteria and subsequent infections. In addition to antibiotics, acid-suppressive medications such as proton pump inhibitors may also influence gut microbiota by altering the gastric acid barrier and have been associated with reduced microbial diversity (Bridgman et al., 2025). Therefore, optimizing nutritional support strategies and minimizing unnecessary antibiotic exposure may represent important approaches for maintaining gut microbial homeostasis in children with SBS.

It is important to note that the mechanisms described above are not mutually exclusive, and the relationships between microbiota dysbiosis, anatomical alterations, nutritional support, and antibiotic exposure are likely bidirectional. Taken together, these interconnected relationships and their potential downstream effects on microbial metabolites, intestinal barrier function, inflammation, and clinical complications are summarized in **Figure 1**.

**Figure 1:**
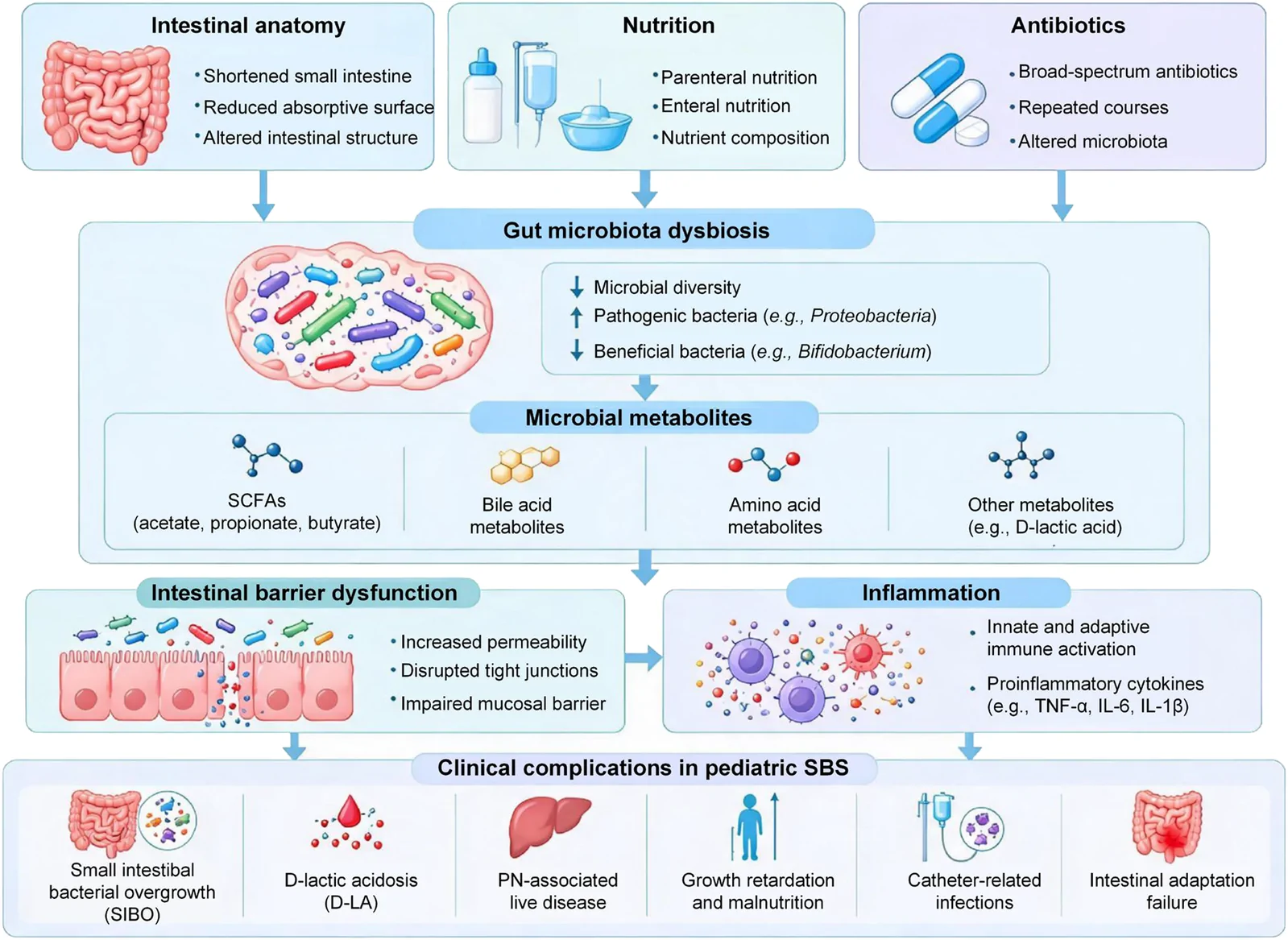
Integrated schematic of the relationships among intestinal anatomy, nutrition, antibiotic exposure, gut microbiota dysbiosis, microbial metabolites, intestinal barrier dysfunction, inflammation, and clinical complications in pediatric short bowel syndrome.

## 5 Gut microbiota and SBS-related complications in children

### 5.1 SIBO

SIBO is one of the most common complications in children with SBS (Zafar et al., 2023). Impaired protective mechanisms, including reduced gastric acid secretion, intestinal dysmotility, and compromised mucosal barrier function, can promote abnormal bacterial proliferation in the small intestine, thereby increasing the risk of SIBO. Clinically, SIBO in children with SBS is characterized by abdominal distension, abdominal pain, increased flatulence, diarrhea, and poor weight gain. In severe cases, SIBO may lead to impaired bile acid and fat absorption, vitamin B_12_ deficiency, and increased risks of bloodstream infections and intestinal failure-related complications (Puoti et al., 2025). A prospective cohort study reported a SIBO prevalence of 66% among children with intestinal failure, and those with SIBO exhibited reduced microbial α-diversity, increased abundance of *Enterobacteriaceae* and *anaerobic bacteria*, and associations with intestinal inflammation, liver injury, and central line-associated bloodstream infections (Hilberath et al., 2025). Another retrospective study demonstrated that SIBO was associated with shorter residual small bowel length and SBS etiology, whereas longer residual bowel length appeared to provide a protective effect (Belza et al., 2020). In children with SBS, recurrent or refractory SIBO may occur and potentially delay the weaning of PN (Velimirovic et al., 2025).

Currently, there is no universally accepted diagnostic criterion for SIBO. Although culture of duodenal or jejunal aspirates has relatively high specificity, its clinical application is limited by invasiveness and the risk of contamination. Breath tests, in contrast, are less invasive and easier to perform. Treatment strategies for SIBO mainly include improving intestinal motility, optimizing nutritional support, and administering intermittent or cyclic antibiotic therapy (Pai and Sainath, 2025). However, long-term repeated prophylactic use of metronidazole and trimethoprim-sulfamethoxazole, although intended to control SIBO, may promote the enrichment of *Proteobacteria* and *Escherichia coli*, further disrupting microbial homeostasis (Phyo et al., 2021). Importantly, changes in relative abundance should not be interpreted as changes in absolute bacterial burden. In SIBO, an increased relative abundance of a bacterial taxon may reflect either expansion of that taxon or a reduction in other bacterial populations. Absolute bacterial quantification is therefore important when interpreting microbial changes in the anatomically altered intestine.

### 5.2 D-LA

D-LA is an uncommon but serious metabolic complication in children with SBS. In children with SBS, impaired carbohydrate absorption results in excessive amounts of unabsorbed carbohydrates reaching the colon, providing fermentable substrates for D-lactate-producing bacteria such as *Lactobacillus*. Moreover, the slower metabolism of D-lactate facilitates its accumulation and contributes to metabolic acidosis (Bianchetti et al., 2018). Clinically, D-LA is mainly characterized by neurological manifestations, including altered mental status, drowsiness, fatigue, gait abnormalities, and slurred speech, while routine lactate measurements may fail to detect this condition (Remund et al., 2023). A case-control study identified midgut volvulus, a history of intestinal lengthening surgery, and elevated anion gap as independent risk factors for D-LA, with midgut volvulus showing the strongest association (Nes et al., 2024). Current treatment strategies for D-LA mainly include restriction of carbohydrate intake, correction of acid-base disturbances, and modulation of abnormal microbiota. Notably, in diarrheic calves, Lactobacillus rhamnosus GG colonized the intestine without altering D-lactate levels (Ewaschuk et al., 2006). Although these veterinary data cannot be directly extrapolated to children with SBS, they suggest that specific probiotic strains may not universally increase D-lactate accumulation.

### 5.3 Enteric-derived infections

Impaired intestinal barrier function and gut microbiota dysbiosis are important contributors to enteric-derived infections in children with SBS. Intestinal dysmotility, bowel dilation, and SIBO may promote the abnormal proliferation of potential pathogens, including *Proteobacteria* and *Enterococcus*, leading to disruption of the mucosal barrier and increased bacterial translocation (Merras-Salmio and Pakarinen, 2022). A study of children with intestinal failure found that catheter-related bloodstream infections were common among those receiving long-term PN, with major pathogens including *Staphylococcus epidermidis*, *Enterococcus faecalis*, and *Klebsiella pneumoniae*, suggesting that enteric-derived pathogens may contribute to infection development (Vicentin de Souza et al., 2026). In addition, excessive bowel dilation may further exacerbate SIBO and microbiota dysbiosis, potentially creating a vicious cycle of microbial imbalance and recurrent infections. Studies have shown that SBS patients receiving home PN with residual small bowel length <50 cm have a higher incidence of catheter-related sepsis, and some causative pathogens originate from enteric Gram-negative bacteria, indicating that bacterial translocation may play a role in SBS-associated infections (Terra et al., 2000).

### 5.4 IFALD

IFALD is a major complication in children receiving long-term parenteral nutrition, and its development is influenced by multiple factors, including prematurity, residual bowel length, duration of PN, and catheter-related bloodstream infections (Mignini et al., 2025). Evidence from pediatric intestinal failure populations suggested that gut microbiota dysbiosis and disruption of the gut-liver axis may represent important links between SBS and IFALD (Jiang et al., 2023). Reduced microbial diversity and enrichment of *Proteobacteria* are often accompanied by impaired intestinal barrier function, facilitating the translocation of pro-inflammatory microbial products such as LPS through the portal circulation, activating hepatic inflammatory pathways including TLR4 signaling, and consequently exacerbating hepatic inflammation, cholestasis, and fibrosis (Lee et al., 2020). Korpela et al. reported that children with intestinal failure exhibited reduced microbial diversity and increased *Proteobacteria* abundance, which were associated with intestinal and hepatic inflammation as well as hepatic steatosis (Korpela et al., 2017). Multi-omics studies have further demonstrated that children with SBS-associated intestinal failure exhibit reduced microbial richness and diversity, with dominant bacterial taxa closely associated with residual bowel length, PN dependence, and bile acid metabolism (Mutanen et al., 2026). In addition, depletion of bile acid-transforming bacteria and impaired FXR signaling may further disrupt bile acid homeostasis and promote IFALD progression (Xiao et al., 2018). Animal studies have also indicated that modulation of gut microbiota can influence bile acid metabolism, intestinal barrier integrity, and hepatic injury (Nagano et al., 2025). Therefore, maintaining microbial homeostasis, preserving intestinal barrier function, and regulating the gut-liver axis may represent important strategies for the prevention and management of IFALD.

## 6 Gut microbiota-targeted intervention strategies

### 6.1 Probiotics and prebiotics

Probiotics may improve SBS-associated microbiota dysbiosis and potentially promote intestinal adaptation through competitive inhibition of pathogenic bacteria, enhancement of intestinal barrier function, regulation of immune responses, and promotion of SCFA production (Stoidis et al., 2011). In animal models of SBS, combined administration of *Lactobacillus* and *Bifidobacterium* has been associated with increased microbial diversity, higher butyrate levels, and improved markers of mucosal adaptation (Pauline et al., 2022). In small pediatric SBS studies,synbiotic formulations containing *Bifidobacterium breve*, *Lactobacillus casei*, and galactooligosaccharides can increase fecal SCFA levels, improve microbial composition, and promote nutritional recovery (Kanamori et al., 2004). In addition, case reports have indicated that probiotics and synbiotics may improve infections and metabolic disturbances by promoting the recovery of beneficial anaerobic bacteria, inhibiting pathogen overgrowth, and reducing PN dependence (Candy et al., 2001). However, clinical evidence regarding probiotic use in children with SBS remains limited. A randomized crossover trial of *Lactobacillus rhamnosus* found no significant improvement in intestinal permeability or stable colonization (Sentongo et al., 2008). In contrast, a randomized trial in children with SBS found that 2 months of supplementation with *Lactobacillus rhamnosus* and *Lactobacillus johnsonii* did not significantly alter fecal microbiota composition, *Lactobacillus* abundance, or growth outcomes (Piper et al., 2020a). Meanwhile, due to impaired intestinal barrier function and increased risk of bacterial translocation in children with SBS, adverse events such as *Lactobacillus* bacteremia and D-LA have been reported (Reddy et al., 2013). The effects of probiotics are likely to be strain-specific, and evidence from one probiotic strain cannot necessarily be extrapolated to others. In children with SBS, particularly those with impaired intestinal barrier function or central venous catheters, probiotic administration should be approached cautiously because of the potential risk of probiotic-associated bloodstream or invasive infections. Current clinical evidence remains limited, and probiotics should therefore not be considered standard therapy for pediatric SBS.

Prebiotics, including galactooligosaccharides, inulin, and dietary fiber, can selectively promote the proliferation of beneficial bacteria and increase SCFA production. Combined administration with probiotics may exert synergistic effects (Ducatelle et al., 2015). However, direct clinical evidence in pediatric SBS remains limited, and differences in residual anatomy, enteral tolerance, and colonic continuity may substantially influence their effects. Thus, current evidence is insufficient to support routine clinical recommendations for prebiotic supplementation in pediatric SBS. Well-designed clinical trials are needed to determine the appropriate substrates, doses, timing, and patient populations most likely to benefit.

### 6.2 Nutritional modulation

Nutritional modulation is a fundamental strategy for improving gut microbiota and promoting intestinal adaptation in children with SBS. Its key principles include early initiation and gradual advancement of enteral nutrition, optimization of nutrient substrate composition, and reduction of PN dependence. In infants and children with SBS,early postoperative enteral feeding, particularly breast milk or amino acid-based formulas, is associated with shorter PN duration and reduced cholestasis severity (Andorsky et al., 2001). Both breast milk and amino acid-based formulas are commonly used in this context. However, their mechanisms may differ. Breast milk provides additional immunomodulatory and microbiota-promoting factors beyond basic nutrition, whereas amino acid-based formulas primarily reduce antigenic load and are used when breast milk is unavailable or poorly tolerated. Evidence from broader pediatric nutrition studies suggests that breast milk may provide additional intestinal protective effects by promoting beneficial bacterial colonization and SCFA production. In SBS patients with preserved colon continuity, fermentable carbohydrates such as starch and pectin can serve as substrates for gut microbiota, promoting SCFA production and improving colonic fluid absorption (Phelps and Warner, 2023). Experimental and pediatric nutrition studies suggest that human milk oligosaccharides, such as 2′-fucosyllactose (2-FL), may also promote intestinal adaptation through microbiota modulation (Mezoff et al., 2016). In addition, diets enriched in n-3 polyunsaturated fatty acids may increase intestinal mucosal mass and surface area, while reducing diarrhea and fat excretion (Yang and Kock, 2010). Therefore, individualized enteral nutrition strategies based on residual intestinal function should be implemented to promote microbial homeostasis and intestinal adaptation.

Currently, microbiota-targeted interventions in children with SBS remain at an exploratory stage. Different probiotics, prebiotics, and nutritional interventions may exert beneficial effects by modulating intestinal microbial composition, improving metabolic function, and promoting intestinal adaptation. Representative studies and their major findings are summarized in **Table 2**.

**Table 2:** Interventions potentially influencing gut microbiota and intestinal adaptation in pediatric SBS.

Type	Study design	Subjects	Intervention	Sampling site/specimen	Main findings	Major limitations	Ref.
Synbiotics	Prospective clinical study	Children with SBS (n=4)	*Bifidobacterium*, *Lactobacillus casei* combined with galactooligosaccharides	Feces; serum	Improved fecal microbiota composition, increased SCFA levels (acetate, propionate, and butyrate), enhanced immunonutritional status, and promoted growth	Very small sample size; prospective uncontrolled design; limited generalizability	(Uchida et al., 2007)
Probiotics	Randomized controlled trial	Children with SBS (n=18)	Daily supplementation with *Lactobacillus rhamnosus* and *Lactobacillus johnsonii* for 2 months	Stool	No significant effects on fecal microbiota composition, *Lactobacillus* abundance, or growth outcomes, suggesting limited efficacy of single-strain probiotics	Small sample size; limited intervention duration; limited evidence for long-term clinical benefit	(Piper et al., 2020a)
Probiotics	Animal study	Neonatal piglet model of SBS	*Lactobacillus* and *Bifidobacterium* probiotics compared with metronidazole and placebo	Colon tissue; stool; colon effluent	Increased microbial diversity, enhanced abundance of *Bacteroidetes* and *Firmicutes*, promoted SCFA production (especially butyrate), and reduced antibiotic-associated dysbiosis	Animal study; limited direct applicability to pediatric SBS	(Pauline et al., 2022)
Probiotic-derived products	Animal study	Rat model of SBS	*Lactobacillus rhamnosus* GG (LGG) or LGG-derived soluble mediators	Ileum tissue; serum	Reduced intestinal permeability and bacterial translocation, attenuated inflammation, enhanced tight junction protein expression, and improved intestinal barrier function	Animal study; mechanisms and effects require validation in pediatric clinical studies	(Wu et al., 2018)
Probiotics	Randomized crossover controlled trial	Children with SBS (n=9)	LGG supplementation for 4 weeks with placebo crossover	Feces; urine; breath	No sustained improvement in intestinal permeability, *Lactobacillus* colonization, or bacterial overgrowth parameters	Very small sample size; short intervention period	(Sentongo et al., 2008)
Nutritional intervention	Clinical cohort study and animal study	Children with SBS (n=55) and animal models	Increased whole-food and dietary fiber intake; assessment of microbiota and bile acid metabolism	Stool/ostomy effluent; plasma	Modulated glycosidase and bile salt hydrolase activity, influenced bile acid metabolism and microbial function, suggesting potential for individualized dietary intervention	Mixed clinical and preclinical evidence	(Kastl et al., 2022)
Nutritional/growth factor intervention	Prospective clinical study	Children with SBS dependent on long-term PN (n=8)	Recombinant human growth hormone (rhGH) treatment for 12 weeks	Blood/plasma; clinical and nutritional assessments	Increased enteral intake, elevated plasma citrulline levels, reduced PN requirements, and promoted intestinal adaptation	Very small sample size; prospective uncontrolled design	(Goulet et al., 2010)
Nutritional metabolic intervention	Animal study	Rat model of SBS	Dietary inositol hexaphosphate supplementation	Feces; serum; intestinal tissue	Increased residual bowel length and mucosal proliferation, reduced intestinal permeability, and promoted intestinal adaptation	Animal study; microbiota-specific effects and clinical applicability remain uncertain	(Wang et al., 2023)

### 6.3 FMT

FMT, which involves transferring gut microbiota from healthy donors into recipients, has recently emerged as a potential microbiota-targeted therapeutic strategy for SBS (Hou et al., 2025). Case reports have indicated that FMT may alleviate recurrent D-LA in children with SBS, suggesting its potential role in correcting microbiota dysbiosis (Davidovics et al., 2017). In a neonatal SBS piglet model with 75% distal small bowel resection, early FMT demonstrated favorable short-term safety and transiently altered gut microbiota composition as well as α- and β-diversity. However, these effects were not sustained over time (Hinchliffe et al., 2022). Furthermore, animal studies have shown that donor microbiota can successfully colonize sham-operated animals but fail to persist in SBS models, suggesting that the SBS-specific intestinal environment may limit the long-term engraftment of exogenous microbiota (Fourati et al., 2023). FMT remains an experimental approach in pediatric SBS, with current evidence derived mainly from case reports and animal studies. Its efficacy and long-term safety have not been established. Potential risks include transmission of pathogens and antimicrobial resistance genes. Donor screening, donor supervision, dosing, route of administration, and monitoring for adverse events are therefore essential considerations before clinical application. FMT should not currently be considered standard therapy for pediatric SBS.

Importantly, the available interventions differ substantially in their evidence level and mechanism. Probiotics and FMT are more directly microbiota-targeted, whereas nutritional interventions, recombinant human growth hormone, and other approaches may influence microbiota indirectly. Therefore, their clinical effects should not be interpreted as equivalent evidence for microbiota-targeted therapy.

## 7 Challenges and future perspectives

Although research on gut microbiota in children with SBS has advanced substantially in recent years, current evidence remains limited by several challenges. Most existing studies are single-center, small-scale, and cross-sectional, with considerable clinical and methodological heterogeneity that limits the comparability and generalizability of findings. Clinical heterogeneity includes differences in age and gestational age, SBS etiology, residual bowel length, ileocecal valve status, colonic continuity, stoma status, enteral nutrition, breastfeeding versus formula feeding, antibiotic exposure, acid-suppressive therapy, hospitalization or intensive care, and underlying disease. In addition, microbiota profiles may vary according to the anatomical site and type of specimen sampled, such as feces, small-intestinal contents, or stoma effluent. Methodological differences, including sequencing platforms, targeted regions, DNA extraction methods, taxonomic databases, and bioinformatic pipelines, may further contribute to inconsistent findings. Therefore, contradictory results should be interpreted in the context of these clinical and methodological variables rather than attributed solely to SBS-associated dysbiosis. The causal relationship between microbiota dysbiosis and intestinal adaptation requires further validation through long-term longitudinal studies. Current studies also primarily rely on fecal samples, and the complex interactions between microbiota and host physiology remain incompletely understood. Clinical evidence supporting microbiota-based interventions, including probiotics, prebiotics, and FMT, remains limited. Future studies should adopt standardized sampling and microbiome analysis protocols, provide detailed clinical and nutritional metadata, and conduct multicenter prospective studies combined with multi-omics approaches to dynamically characterize microbiota evolution and identify potential biomarkers, followed by randomized controlled trials to evaluate microbiota-targeted interventions in pediatric SBS.

## 8 Conclusion

Children with SBS exhibit significant gut microbiota dysbiosis, with altered intestinal microbial composition closely associated with intestinal barrier impairment, nutritional support, and antibiotic exposure, and potentially contributing to complications such as SIBO, D-LA, and liver injury. Microbiota-targeted strategies, including probiotics, prebiotics, FMT, and nutritional modulation, show potential benefits, but their efficacy and safety require further validation. Future multi-omics and longitudinal studies are needed to clarify microbiota-host interactions and develop effective interventions to improve intestinal adaptation and long-term outcomes in children with SBS.
